# The use of Dose Management Systems in Europe: Results of an ESR EuroSafe Imaging Questionnaire

**DOI:** 10.1186/s13244-024-01765-x

**Published:** 2024-08-09

**Authors:** Reinhard Loose, Eliseo Vaño, Josefin Ammon, Jonas Andersson, Hugues Brat, Boris Brkljacic, Katrina Caikovska, Riccardo Corridori, John Damilakis, Timo De Bondt, Guy Frija, Claudio Granata, Christoph Hoeschen, Elmar Kotter, Ivana Kralik, Jonathan McNulty, Graciano Paulo, Virginia Tsapaki, on behalf of the European Society of Radiology

**Affiliations:** 1 Institute of Medical Physics, Paracelsus Medical School, Hospital Nuremberg, Nuremberg, Germany; 2 Radiology Department, Complutense University, Madrid, Spain; 3 Department of Diagnostics and Intervention, Radiation Physics, Umeå University, SE-091 87 Umeå, Sweden; 4 Swiss Imaging Network, Sion, Switzerland; 5 University of Zagreb School of Medicine, Department of Diagnostic and Interventional Radiology, UH Dubrava, Zagreb, Croatia; 6 Children’s Clinical University Hospital, Riga, Latvia; 7 European Coordination Committee of the Radiological, Electromedical and Healthcare IT Enterprises (COCIR), Brussels, Belgium; 8 University of Crete, School of Medicine, Iraklion, Crete, Greece; 9 Department of Medical Physics, VITAZ, Moerlandstraat 1, 9100 Sint-Niklaas, Belgium; 10 Paris Cité University, Paris, France; 11 Department of Pediatric Radiology Institute for Maternal and Child Health—IRCCS “Burlo Garofolo”—Trieste (I), Trieste, Italy; 12 Otto-von-Guericke University Magdeburg, Magdeburg, Germany; 13 Department of Radiology, Medical Center—University of Freiburg, Faculty of Medicine, University of Freiburg, Freiburg, Germany; 14 School of Medicine, University College Dublin, Dublin, Ireland; 15 Health and Technology Research Center, Escola Superior de Tecnologia da Saúde de Coimbra, Instituto Politécnico de Coimbra, Coimbra, Portugal; 16 Konstantopoulio General Hospital, Athens, Greece; 17 Am Gestade 1, 1010 Vienna, Austria

**Keywords:** Radioprotection, Computer applications-general, Diagnostic procedure, Quality assurance, Dose management

## Abstract

**Abstract:**

Dose management systems (DMS) are an essential tool for quality assurance and optimising patient radiation exposure. For radiologists and medical physicists, they are important for managing many radiation protection tasks. In addition, they help fulfil the requirements of Directive 2013/59/EURATOM regarding the electronic transmission of dosimetric data and the detection of unintended patient exposures. The EuroSafe Imaging Clinical Dosimetry and Dose Management Working Group launched a questionnaire on the use of DMS in European member states and analysed the results in terms of modalities, frequency of radiological procedures, involvement of medical physics experts (MPEs), legal requirements, and local issues (support by information technology (IT), modality interfaces, protocol mapping, clinical workflow, and associated costs).

**Critical relevance statement:**

Despite the great advantages of dose management systems for optimising radiation protection, distribution remains insufficient. This questionnaire shows that reasons include: a lack of DICOM interfaces, insufficient harmonisation of procedure names, lack of medical physicist and IT support, and costs.

**Key Points:**

- Quantitative radiation dose information is essential for justification and optimisation in medical imaging.
- Guidelines are required to ensure radiation dose management systems quality and for acceptance testing.
- Verifying dose data management is crucial before dose management systems clinical implementation.
- Medical physics experts are professionals who have important responsibilities for the proper management of dose monitoring.

**Graphical Abstract:**

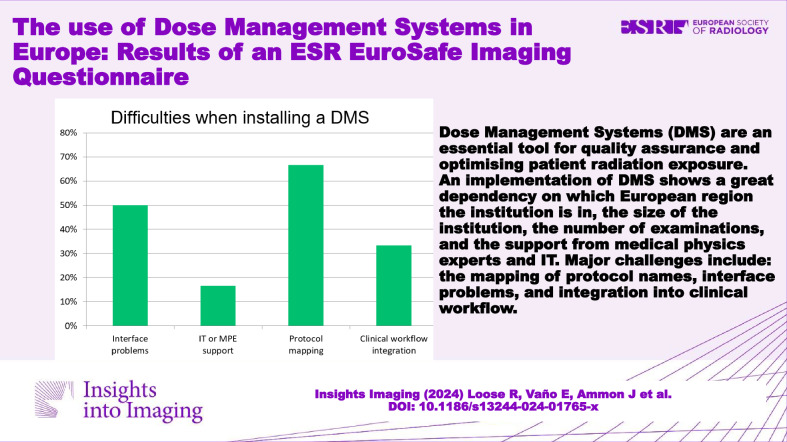

## Introduction

The collection, processing, and evaluation of exposure data from medical procedures involving ionising radiation is an essential part of medical radiation protection. This information is important in many ways, including the safety of patients, optimisation of procedures, establishing of local and national diagnostic reference levels (DRLs), detecting of unintended patient exposures, and radiation protection in the context of medical research involving ionising radiation. Other aspects include replacing manual dose recording, dose documentation, dose reporting, tracking, and analysing large exposure databases. Following the recommendations of the EuroSafe Imaging Steering Committee, two working groups (WG) were established. One on the topic “dosimetry for imaging in clinical practice” and one on the topic “dose management systems (DMS)”. The results and recommendations of both working groups are intended, among other things, to help EU Member States (EU-MS) implement and harmonise Directive 2013/59/EURATOM (EU-BSS) [1] on essential radiation safety standards. Both working groups produced several publications and scientific posters on collecting and processing clinical dosimetric data and DMS [2–4]. In addition, the results of a survey in EU-MS on the scope and difficulties in implementing the EU-BSS were published [5]. In 2022, both WGs were merged into a joint WG on Clinical Dosimetry and Dose Management. The new WG’s first aim was to create a questionnaire to evaluate the spread of DMS installations among EU-MS and associated difficulties with modality interfaces, staff qualifications, financial resources, and national regulations.

The International Atomic Energy Agency (IAEA) published many aspects of medical exposure monitoring in the comprehensive “Safety Reports Series 112: Patient Radiation Exposure Monitoring in Medical Imaging” without focusing on implementation and difficulties with DMS [6].

There is consensus (also between the WG and IAEA) that modality-related dosimetric parameters should be used to assess patient exposure [2, 6, 7]. The most relevant radiological parameters, quantities, and units are:

- Kerma area product for Radiography, dental radiography, and cone beam CT *P*_KA_ (mGy·cm^2^)
- Kerma area product for Fluoroscopic procedures *P*_KA_ (Gy·cm^2^)
- Air kerma at the patient entrance reference point for fluoroscopic procedures *K*_a,r_ (mGy)
- Volume CT air kerma index for Computed Tomography CTDI_vol_ (mGy)
- Air kerma length product for computed tomography dose length product (mGy·cm)
- Average glandular dose in Mammography AGD (mGy)

The DICOM (Digital Imaging and Communications in Medicine) standard was established worldwide for the electronic transfer of images and dosimetric data. DICOM images contain only exposure data of single images in their headers, but a procedure’s cumulative exposure is missing. In particular, the exposure of fluoroscopic procedures without exposed single images or series needs to be included. The DICOM Radiation Dose Structured Report (RDSR) contains a list and sum of all exposures of an individual procedure, which fulfils an important function here. In multislice CT, RDSR includes the dose contribution of overbeaming and over-ranging outside a series of reconstructed images. All modalities transfer their DICOM dosimetric data to one or multiple destinations like radiology information system (RIS), picture archiving and communication system (PACS), or a DMS. In addition, some DMS manufacturers also calculate effective doses or organ doses for individual patients. These calculations should be evaluated with caution as they may involve large uncertainties. The ICRP, therefore, does not recommend using the effective dose for individual patient examinations [8, 9]. The effective dose is used to assess the risk of occupational exposure, for unintentional exposure of patients and pregnant women, and for dose comparison of procedures with different dose units, e.g., radiology and nuclear medicine or CT and radiography. The aim of the WG, with this survey among European Society of Radiology (ESR) members in European Union (EU) member states, was to gain an overview of the implementation and associated difficulties in planning and using DMS.

## Dose management systems (DMS)

Dose management systems (DMS) were introduced to the market more than ten years ago and in the meantime have become more widespread, particularly in larger radiological facilities. They are an essential tool for quality assurance and optimising patient radiation exposure. For radiologists and medical physicists, they are an important tool for managing many radiation protection tasks. In particular, the requirements of the EU BSS [1] about the electronic transmission of dosimetric data (EU-BSS Art. 60, 3. (c)) and the detection of unintended patient exposures (EU-BSS Art. 63 (b)) [1, 4] have contributed significantly to the spread of DMS in Europe. However, it turns out that the frequency of successful DMS installations depends on various factors. Among other things, these are national legal requirements, suitable interfaces to the modalities, harmonised examination protocols, the size of departments, the integration into existing IT infrastructure, and in particular, the availability and involvement of medical physicists (medical physics experts, MPEs). The complexity of a successful implementation depends on the modality used. Fortunately, this is easiest for CT with the highest collective population exposure. All CT systems provide reliable interfaces with DICOM image data with dose information, and many systems provide a standardised RDSR [10, 11]. In 2022, the EuroSafe Imaging Clinical Dosimetry and Dose Management WG launched a questionnaire on the implementation, use, and difficulties of implementing a DMS among EuroSafe Imaging Stars (IS) in European Member States and other individual members of the ESR. The analysis of answers in this publication shows considerable heterogeneity about the parameters mentioned and a bias about the frequency of answers from DMS users and DMS-non-users.

## Methods

The questionnaire was coordinated in the WG with regard to the number and details of the questions and was created using the SurveyMonkey tool. Each question allowed between 4 and 7 multiple choice answers, some included additional free text. Table 1 shows the 16 questions (with minor modifications for better readability).

**Table 1:** List of the 16 Survey Monkey questions without multiple-choice answer checkboxes and free text fields

1. Country of workplace
2. Your role in the facility?
3. Number of radiological procedures/year with ionising radiation, including interventions
4. Number of modalities in your facility
5. If you don’t use a DMS, what are the main reasons?
6. Number of modalities sending dosimetric DICOM data to your DMS
7. Main difficulties and expenditure of personnel or time when installing a DMS
8. Does your facility provide dosimetric information about individual patient exposure to users, referrers, or patients?
9. Are you reporting individual patient exposures in values and units reported by the modalities?
10. Do you think reporting of individual patient exposures to destinations could be solved with a DMS?
11. Are you or your facility following the specific recommendation: “If an accidental or unintended individual patient exposure is suspected, the dose parameters (based on physical quantities) should be recorded, analysed, and a report prepared for the required committee or authority”?
12. Do you think managing unintended exposures could be solved with a DMS?
13. In your facility, do you compare patient exposures with national and/or local diagnostic reference levels (DRL), e.g., at periodic intervals or after changes in an X-ray modality or imaging protocols?
14. Do you think comparing patient exposures should be solved with a DMS?
15. Do you feel setting up local and/or clinical DRLs could be solved with a DMS?
16. Could you list the main problems identified in your facility to fulfil the European Directive 2013/59/EURATOM during the implementation period?

The questionnaire was sent to a random selection of five ESR members per country (244), as well as all IS facilities (128). In summary, the questionnaire was sent to a total of 372 recipients. After the initial call before the European Congress of Radiology (ECR) 2022 and a reminder after ECR 2022, 60 replies (16%) were received, and 56 responses could be evaluated without missing data. The questionnaire contained yes/no questions, multiple choice questions, and questions with free text answers. The analysis, conclusions, and recommendations of this EuroSafe Imaging [12] questionnaire shall help operators of X-ray modality optimise patients’ radiation exposure and implement the EU-BSS requirements [1, 2, 4].

## Results

### Where did the answers come from?

**Fig. 1:**
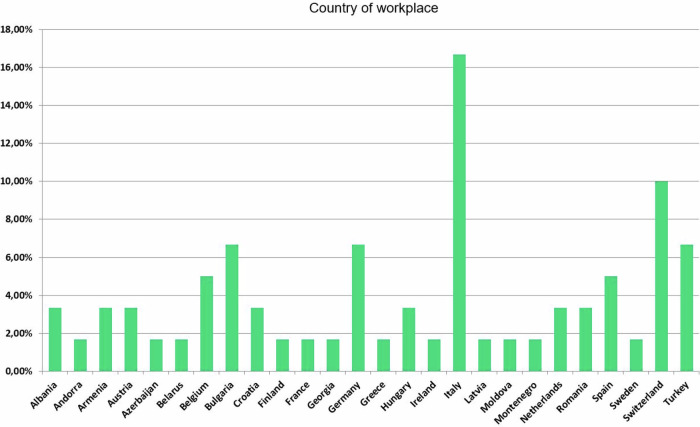
Relative frequency of questionnaire responses from different countries

Figure 1 shows the relative frequency of replies from 26 different countries, with the highest participation from Italy. The answers showed clear differences in the analysis of individual subgroups, and a bias in the frequency of responses from IS compared to non-imaging stars (NIS). Regarding geographical location, 62% of DMS users came from Western European countries, while 38% were from Eastern European countries. 38/56 (68%) answers came from IS, but only 18/56 (32%) from NIS. The results regarding the geographical region and the “Imaging Stars” certificate are not surprising. It can be assumed that fewer DMS are installed in Eastern Europe than in Western Europe, and facilities that have acquired an IS certificate are more likely to operate a DMS and respond more frequently. Overall, this is an unavoidable bias in the survey that a DMS user is more likely to answer than a non-user.

### Who answered the questionnaire?

**Fig. 2:**
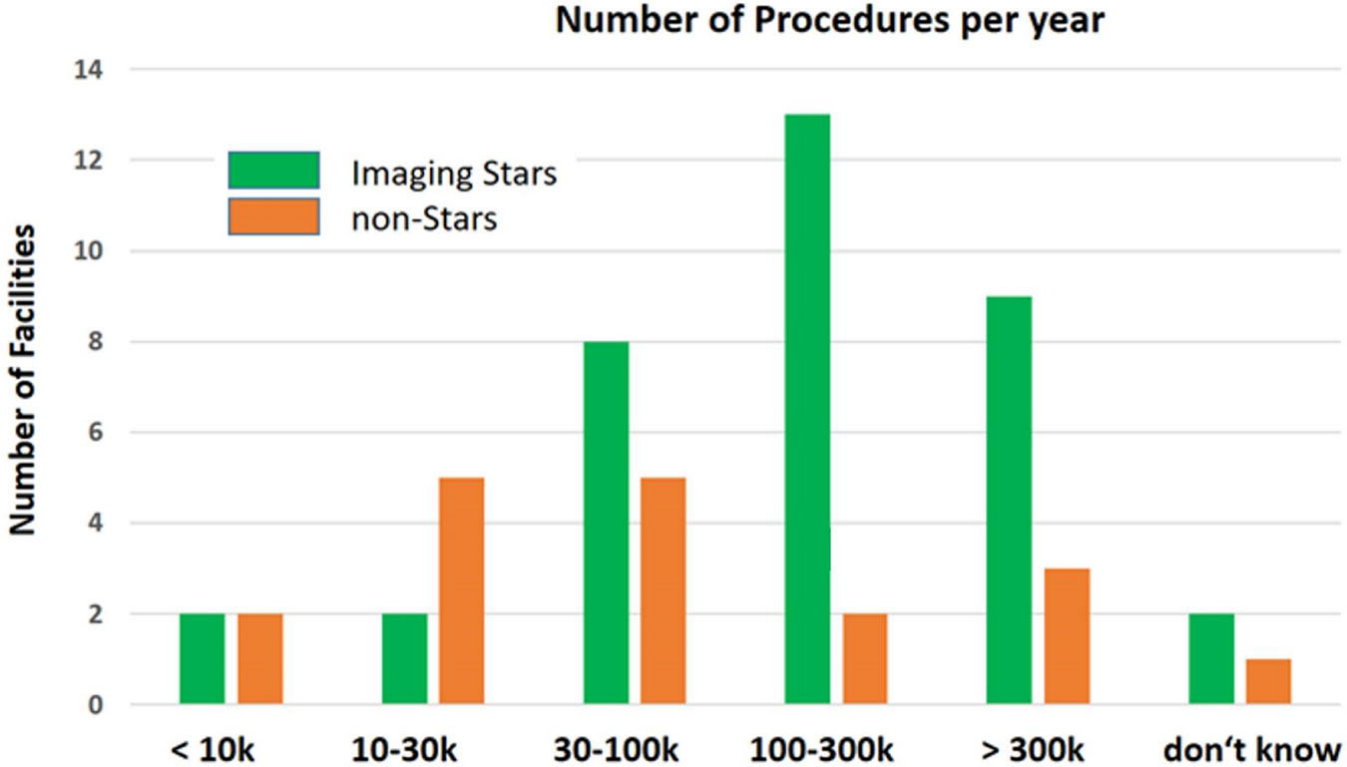
Average number of procedures/year performed in different facilities

For IS facilities, 51% of responses came from MPEs and 26% from radiologists. For NIS facilities, 16% of responses came from MPEs and 72% from radiologists. This clearly shows that IS have higher availability and involvement of MPEs. 82% of IS use a DMS, but only 28% of NIS. Most DMS users reported exam frequencies from 100,000 to over 300,000 per year, while non-DMS users reported much lower frequencies between 10,000 and 30,000 per year (Fig. 2). In summary, the likelihood of a DMS installation increases with increasing annual examination frequency and higher availability of MPEs.

### What are the reasons for not having a DMS installed?

**Fig. 3:**
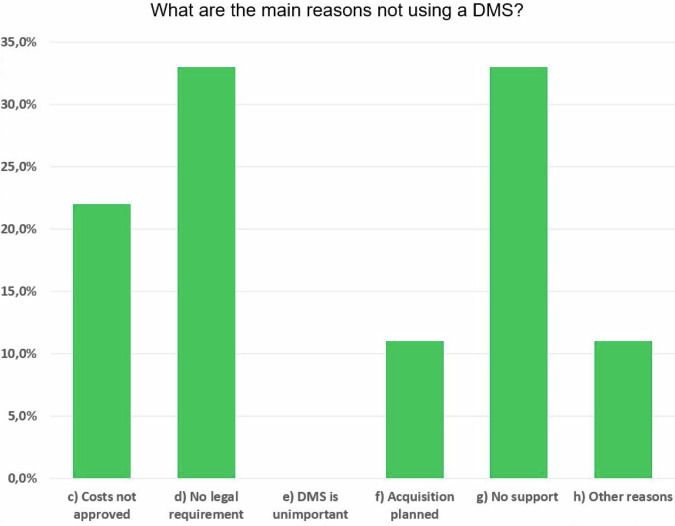
Main reasons of DMS non-users why a DMS is not installed

Figure 3 shows the most common reasons facilities did not have a DMS installed. The three main reasons were lack of support, no legal requirements, and no approval of the costs. It is correct that the EU-BSS does not require a DMS but only the electronic transmission of exposure data. Nevertheless, in medium-sized and larger facilities, MPEs or radiologists will hardly be able to meet exposure monitoring requirements without a DMS. The costs of a DMS are likely to play a role, especially in smaller facilities, as many DMS are offered as a local installation with server hardware and software at high prices. In particular, web-based solutions that transfer the exposure data to a cloud server for storage and evaluation require only minimal local hardware and can be operated inexpensively as a “pay-per-use” model.

### What are the difficulties with DMS installation?

**Fig. 4:**
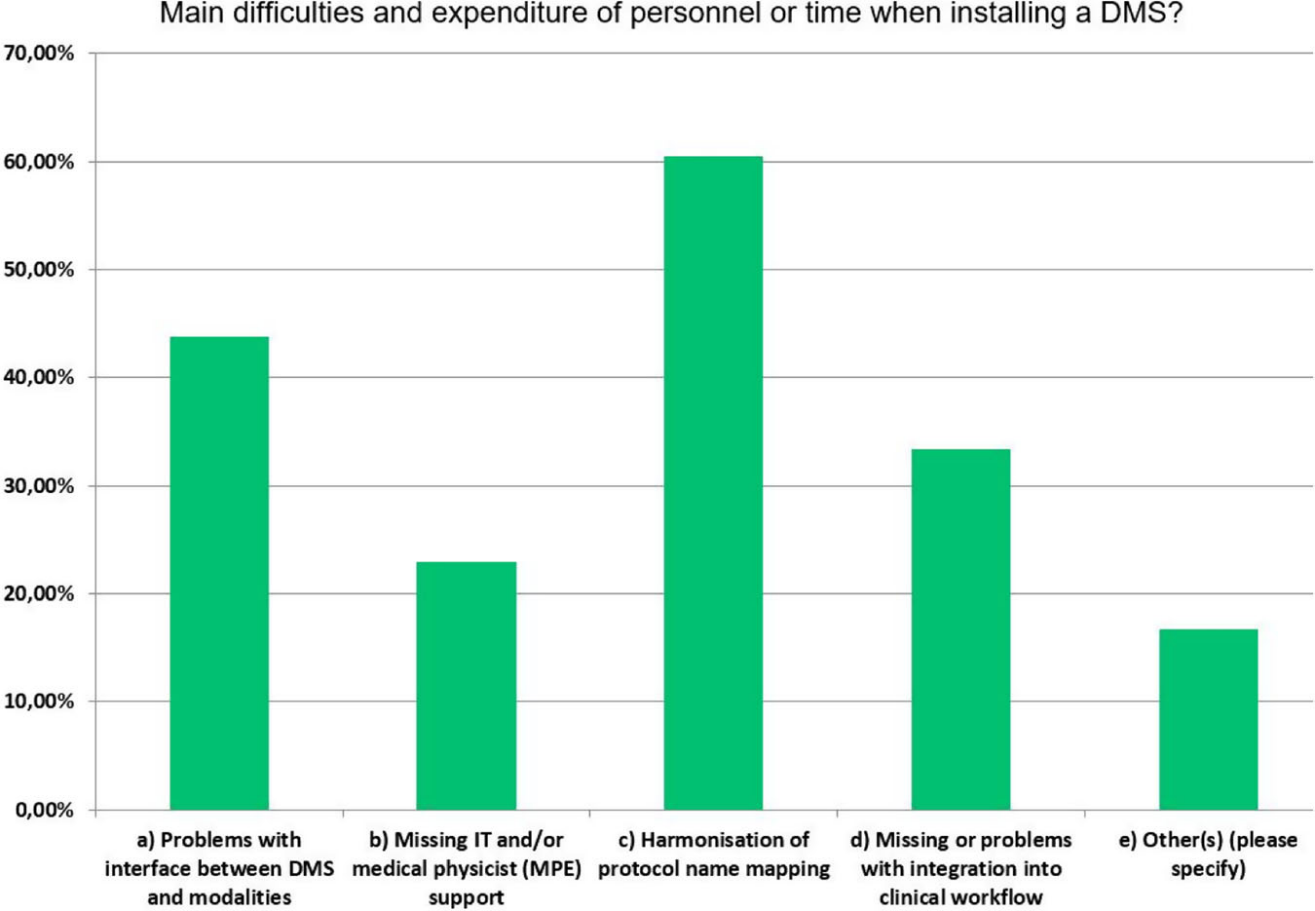
Reported main difficulties of DMS users when installing their DMS

Figure 4 shows the main challenges identified with installing a DMS. The most commonly reported problems were the harmonisation of protocol names for mapping, followed by issues with the interface between DMS and modalities, problems with integration into clinical workflow, and missing IT and/or MPE support. The need to harmonise protocol names is the most frequently mentioned problem after installing a DMS. This harmonisation would enable benchmarking of exposure data at local, regional, national, and international levels and its use for optimisation and creating local or national DRLs [13]. Such harmonisation is necessary for meaningful evaluations to be possible locally within a facility. At a large hospital (Paracelsus Medical School, Hospital Nuremberg, Germany), there are around 100 radiological procedures that clinicians can request. From these, around 1000 different protocols are generated for individualised diagnostic examinations and interventional procedures. Unfortunately, this situation exists in many countries and facilities, so standardisation of the examination protocols such as RadLex or LOINC [14, 15] would be urgently needed. Unfortunately, many countries have no binding requirements for using standardised protocol names.

The second problem is insufficient digital interfaces to modalities. Nearly all new radiological modalities have implemented the DICOM standard and RDSR and thus provide dosimetric data at the level of individual images or for entire procedures. This is not the case for many old devices, for example, old fluoroscopy C-arms in operating rooms, but it can be assumed that such devices will disappear from the market over the next few years.

### Typical DMS applications

Eighty-six percent of responders said they verify exposure data against national DRLs, but only 42% against local DRLs. The highest frequency of modalities sending dosimetric data to a DMS was CT (96%), followed by interventional fluoroscopy (82%), mammography (80%), diagnostic fluoroscopy and radiography (78%), and positron emission tomography (PET)-CT/single-photon emission computed tomography (SPECT)-CT (62%). Dose data were provided to patients (only on request) (38%), in the medical patient report (36%), in the internal patient record (30%) and to the referrer (8%). Only 30% of individual patient exposures were reported to the patient, medical report to the referrer, or internal patient record in the values and units reported by the modalities [2, 3, 5]. Most respondents (71%) indicated that the issues around reporting individual patient exposures could be solved with a DMS. For CT, the most common modality to report data to a DMS, 84% of respondents thought setting up local and/or clinical DRLs could be solved with a DMS.

Tsalafoutas et al [16] published the results of a questionnaire among manufacturers of DMS, which focused exclusively with yes/no answers on manufacturers’ specifications. The results of the survey are also very heterogeneous. Positive answers (mean values) relate to data transfer from the modalities with 80%, data collection with 76%, statistical analysis with 69-100%, customising the DMS software with 59% and service and support with 59%. The results clearly exhibit that large differences exist between the various DMS vendors.

Available DMS features such as calculating cumulative patient doses over multiple exams and a longer period of time are also offered but require national or regional exposure databases [17].

In this EuroSafe Imaging survey among institutions operating a DMS, the problem of harmonising protocol names was frequently mentioned at over 60%. Within a single facility, the lack of harmonisation of protocol names is less of a problem if identical protocol names are used when multiple modalities are used for the same procedure. However, comparisons at regional or national level are only possible if the local protocol names are converted into uniform protocol names using translation tables. Even the creation of such tables is often ambiguous and challenging because local protocol names often contain complex ranges of anatomical regions and examination strategies. To create national DRLs, at least unique protocol names that can be mapped to the national nomenclature are required.

The costs, the time required for radiologists, MPEs, and IT staff, as well as the complexity of the hardware and software to be installed, are limiting factors, especially for small institutions. Many DMS are offered as a local installation with server hardware and software at high prices. In particular, web-based solutions that transfer the exposure data to a cloud server for storage and evaluation require only minimal local hardware and can be operated inexpensively as a “pay-per-use” model. In decreasing order of availability, dosimetric data from CT, interventional devices, mammography, diagnostic fluoroscopy, radiography, and PET-CT can be transmitted electronically to subsequent systems. However, successful installation in a clinical environment requires a clinical information system with at least an health level seven interface, an RIS with a DICOM work list, and a PACS. Only then can exposure data be transferred to the medical record and in the report to the referrer and patient.

To succeed in a DMS installation, a focus on protocol harmonisation (particularly based on clinical indications in CT examinations), leveraging RadLex through translation tables, is recommended as a first step. This should involve regular meetings and active collaboration between radiologists, radiographers, medical physics experts, and field engineers. Each of them needs a certain percentage of their working time to be allocated to DMS related projects. Additionally, communicating results to higher hospital management will help with the justification of utilising resources for the DMS. Regular review and optimisation of protocols based on DMS data are advised [13]. IT interoperability is essential and including a specialised IT technician is crucial to ensure adequate system integration and operation.

## Conclusions

DMS have proven to be an essential tool for collecting, storing, and analysing medical patient exposures. So far, DMS have helped optimise the radiation protection of patients, the analysis of individual and population-based exposures, and the process of justification and optimisation of protocols. Information on patient exposure at the population level is informative for assessing trends in collective doses, setting up clinical, local, and national DRLs, and as a basis for epidemiological studies on the effects of radiation. The technological developments of radiological modalities have improved electronic access to information on patient exposure and the analytical uses of these data [6]. This study aimed to obtain information about reasons for or against the installation of DMS depending on the geographical location of EU member states and the size of radiological facilities.

According to the questionnaire respondents, IS hospitals have a significantly higher number of installed DMS and a greater participation of MPEs than other facilities. Furthermore, CT was the most commonly connected modality to a DMS. The reasons for not having DMS installations included unapproved costs and no implementation support. Inadequate harmonisation of protocol names remains a major challenge. Facilities planning the acquisition of a DMS should carefully explore the available DMS solutions, regarding their features and functionalities, to make sure that they meet their specific needs.

The next planned step of the WG is to propose recommendations on best practices for DMS installations, especially in smaller facilities.

## Data Availability

Summary data from the survey is available in tables within the manuscript. Upon reasonable request, further data can be made available from the corresponding author.

## Abbreviations

AGD: Average glandular dose
CT: Computed tomography
CTDIvol: Volume computed tomography dose index
DICOM: Digital imaging and communication in medicine
DMS: Dose management systems
DRL: Diagnostic reference level
ECR: European Congress of Radiology
ESR: European Society of Radiology
EU: European Union
EU-MS: EU member states
IAEA: International Atomic Energy Agency
ICRP: International Commission on Radiological Protection
IS: Imaging star
IT: Information technology
MPE: Medical physics expert
NIS: Non-imaging star
PACS: Picture archiving and communication system
PET: Positron emission tomography
RDSR: Radiation dose structured report
RIS: Radiology information system
WG: Working group
